# Efficacy and safety of foam sclerotherapy for great saphenous vein reflux: a systematic review and meta-analysis of RCTs

**DOI:** 10.3389/fmed.2026.1903287

**Published:** 2026-09-14

**Authors:** Junchen Wang, Xiaojuan Kou, Yunyun Shuai, Fan Wang, Wei Hu, Gang Zhou, Zhaoxia Li

**Affiliations:** 1 The Second Hospital of Gansu Province, Lanzhou, Gansu, China; 2 School of Clinical Medicine, Northwest Minzu University, Lanzhou, Gansu, China; 3 Department of Cardiology, Gansu Provincial Hospital, Lanzhou, Gansu, China; 4 The First School of Clinical Medicine, Lanzhou University, Lanzhou, Gansu, China

**Keywords:** foam sclerotherapy, great saphenous vein reflux, meta-analysis, randomized controlled trials, systematic review

## Abstract

**Objective:**

This systematic review and meta-analysis compared the efficacy and safety of foam sclerotherapy vs. HL/S for great saphenous vein (GSV) reflux.

**Methods:**

We searched eight databases from inception to July 2026 for randomized controlled trials (RCTs) of foam sclerotherapy for GSV reflux. Risk of bias was assessed using the Cochrane risk of bias tool. Random-effects meta-analyses were performed to pool the effects for each outcome with the longest follow-up time, and the Grading of Recommendations, Assessment, Development and Evaluations (GRADE) approach was used to rate the certainty of evidence.

**Results:**

Twenty-three trials (3,122 patients) were included. Foam sclerotherapy may provide benefits over HL/S for symptom improvement (low certainty: RR, 1.09, 95% CI, 1.03–1.16) and several perioperative outcomes (low to very low certainty), including shorter surgery duration (WMD −35.28 min), reduced hospital stay (WMD −3.72 days), lower costs (WMD −4622.34€), and less blood loss (WMD −50.48 mL). However, foam sclerotherapy had a significantly lower occlusion rate (low certainty: RR, 0.65, 95% CI, 0.49–0.86), and no statistically significant difference was observed in recurrence rates (very low certainty: RR, 0.56, 95% CI, 0.25–1.23).

**Conclusions:**

Foam sclerotherapy may yield better symptom improvement, safety, and perioperative outcomes than HL/S for GSV reflux despite a lower occlusion rate. While comparable recurrence rates were noted between treatments, this result does not confirm equivalent long-term durability of the two interventions. The overall certainty of evidence was low, warranting further high-quality standardized long-term studies.

**Systematic Review Registration:**

https://www.crd.york.ac.uk/PROSPERO/view/CRD42024537972, identifier [CRD42024537972].

## 1 Introduction

Great saphenous veins (GSV) reflux are a prevalent vascular disorder with multiple causes, including genetic predisposition, lifestyle factors, and occupational influences (1). The global prevalence of this condition varies substantially, ranging from 2% to 73% depending on demographic and geographic characteristics (2, 3). In addition, GSV reflux heavily burden the healthcare system due to the associated healthcare costs and lost productivity (4).

Historically, high ligation and stripping (HL/S) was the mainstay of treatment for GSV reflux (5). In recent years, foam sclerotherapy has emerged as a minimally invasive, cost-effective, and safe alternative in clinical practice. However, the long-term occlusion rate of foam sclerotherapy for GSV reflux is debated, with the literature showing 1-year occlusion rates of up to 85%–95% (6–8), but recurrence rates are higher at 5 years, more than 30% (9, 10).

Several systematic reviews and meta-analyses have investigated the management of GSV reflux (11–17). However, these studies have certain limitations, including insufficient evaluation of evidence certainty (11–17), incomplete search strategies (17), inadequate inclusion/exclusion criteria (11, 12, 14, 17), lack of pre-registered protocols (11, 12, 14, 17), failure to explore heterogeneity in pooled effects estimates (12, 15, 17). To address these limitations, we conducted a systematic review and meta-analysis to assess the efficacy and safety of foam sclerotherapy for the GSV reflux.

## 2 Methods

This systematic review was reported according to the Preferred Reporting Items for Systematic Reviews and Meta-analyses (PRISMA) statement (18). We registered the protocol on the PROSPERO (CRD42024537972).

### 2.1 Search methods

We conducted electronic searches in the Embase, PubMed, Web of Science, Cochrane Library, China National Knowledge Infrastructure (CNKI), Wanfang Database, China Science and Technology Journal Database (CQVIP), and Chinese Biology Medicine Disc (CBMdisc) from inception to July 2026. The main search terms included “sclerotherapy,” “saphenous,” and “varicose veins”. Additionally, we manually searched the reference lists of included studies and previous systematic reviews to identify potentially eligible studies. No restrictions were applied on publication time or language. The search strategy for each database is presented in the Supplementary materials search strategy.

### 2.2 Eligibility criteria

We included RCTs of patients with GSV reflux confirmed by color Doppler ultrasound, comparing foam sclerotherapy with HL/S. Studies using HL/S combined with other venous therapies were excluded. No restrictions were applied regarding the follow-up duration.

#### 2.2.1 Outcome measures

Primary outcomes included:

- Occlusion rate: complete GSV occlusion at mid-thigh level with absence of flow/reflux;
- Recurrence rate: recurrence of varicose veins or symptoms with duplex-confirmed reflux/recanalisation (typically reflux >0.5 s), as defined by each trial;
- Symptom improvement: after treatment, patients showed positive improvement in symptoms such as hyperpigmentation, dermatitis, and ulcers (this definition is summarized from the content defined by the author in the article).

Secondary outcomes included:

- Operative parameters: including procedure duration and blood loss.
- Healthcare utilization: including hospital stay duration (days) and costs.
- Patient-reported outcomes: including satisfaction surveys and quality of life scores.
- Venous Clinical Severity Score (VCSS).
- Adverse events: including thrombophlebitis, hyperpigmentation, infections, haematoma and others.

Exclusion criteria:

We excluded case reports, duplicate publications, conference abstracts, editorials, and ongoing trials.

### 2.3 Study selection

Three reviewers (X.K., J.W., Y.S) independently screened titles and abstracts of all citations searched and full texts of potentially eligible studies. Disagreements were resolved through consensus discussion or, when necessary, by consultation with a fourth reviewer's (Z.L or G. Z.).

### 2.4 Data extraction

Using standardized, pilot-tested forms, two reviewers (F.W., Y.S.) independently extracted data from each eligible trial in duplicate, including study's characteristics (e.g., first author's name, publication year, study location/countries, funding source, and trial registration), patient characteristics [e.g., age, sex, clinical etiology anatomic pathophysiologic (CEAP) clinical classification stages, disease course], treatment details (e.g., sclerosant type and dosage, treated vein diameter and compression therapy protocol (including stocking use and duration), and all predefined primary and secondary outcomes with their respective follow-up duration. When outcomes were reported as ordinal scales (e.g., significant, effective, and ineffective), we dichotomized the results into significant/effective vs. ineffective categories. All hospitalization costs were converted to euros (EUR) using the exchange rates from each study's publication year, without inflation adjustment.

### 2.5 Risk of bias assessment

Pairs of reviewers (Y.S., X.K.) independently assessed the risk of bias of eligible studies using Cochrane risk of bias instrument (19). Risk of bias was reported using RevMan v5.3 for randomized sequence generation of included studies, allocation concealment and blinding of outcome assessors, incomplete outcome data (≥20% missing data were considered high risk of bias), and selective reporting.

### 2.6 Statistical analyses

All meta-analyses were performed using Stata version 17 (StataCorp). For dichotomous outcomes, we calculated risk ratios (RR) with 95% confidence intervals (CI). For continuous outcomes, weighted mean differences (WMD) with 95% CIs were adopted to pool data if the same measurement scale was applied across studies; standardized mean differences (SMD) with 95% CIs were used when outcome assessment tools or measurement units differed substantially among included trials (20, 21). Random-effects models were used for all meta-analyses due to expected high heterogeneity among trials (22). In cases of zero-event studies, we applied the Mantel-Haenszel method with a 0.5 continuity correction and followed established zero-event frameworks for classification (23).

For multi-arm randomized controlled trials sharing a single common comparator arm, we applied the control-arm splitting technique to mitigate unit-of-analysis bias. All intervention arms meeting our predefined inclusion criteria were preserved to enable separate effect estimates; the total sample size and event numbers of the shared control group were divided equally proportional to the number of eligible intervention arms, and each subdivided control cohort was paired with one intervention arm to form independent two-arm comparisons for pooled analysis. Any intervention arms unrelated to the primary comparison framework were excluded during data extraction and quantitative synthesis (24).

Prespecified subgroup analyses examined potential effect modifiers, including: (1) publication language (Non-English vs. English), which was selected owing to well-documented language bias that non-English trials tend to overestimate pooled effect sizes (25); and (2) compression therapy regimen (treatment group alone vs. both groups wearing compression socks), predefined as an *a priori* moderator because compression socks independently improve postoperative recovery, and whether compression is applied to the control arm may alter the observed between-group treatment difference; (3) follow-up duration, predefined as an *a priori* moderator, since the therapeutic efficacy of venous interventions differs significantly across different follow-up durations.

The primary meta-analysis pooled all data from single and repeated foam sclerotherapy sessions. A sensitivity analysis limited to studies implementing repeat treatment was conducted to test the stability of the pooled effect sizes; we additionally performed sensitivity analysis excluding high risk of bias RCTs to evaluate the robustness of our findings. We further applied the Hartung-Knapp-Sidik-Jonkman method (26) to evaluate the robustness of pooled effect estimates. Publication bias was assessed through visual inspection of funnel plots for meta-analyses including ≥ 10 studies.

### 2.7 Certainty of evidence

The certainty of evidence for each outcome was evaluated using the Grades of Recommendation, Assessment, Development, and Evaluation (GRADE) approach (27). We assessed five key domains: risk of bias, inconsistency, indirectness, imprecision, and publication bias, grading the evidence as high, moderate, low, or very low.

Judgements for inconsistency were based on the I² statistic, alongside comprehensive assessment of confidence-interval overlap, magnitude and direction of effect, and clinical heterogeneity across studies. Imprecision was comprehensively assessed considering confidence interval width, total sample size and total number of events. The certainty of evidence was rated independently for each outcome. The 95% prediction intervals calculated in this study only served as auxiliary references for interpreting inconsistency and imprecision (28). The certainty of evidence could be downgraded by one or two levels according to the severity of study limitations. Two independent reviewers (F.W., H.W.) completed all GRADE certainty assessments, and any disagreements were resolved through consensus.

## 3 Results

### 3.1 Study selection

Of 7,001 citations, 23 eligible trials involving 3,122 patients were included in the review. Including 5 English-language trials [9 publications (10, 29–36) with 4 trials published twice with different follow-up times (10, 29, 31–36)], and 18 Chinese-language trials (37–54) (Figure 1).

**Figure 1:**
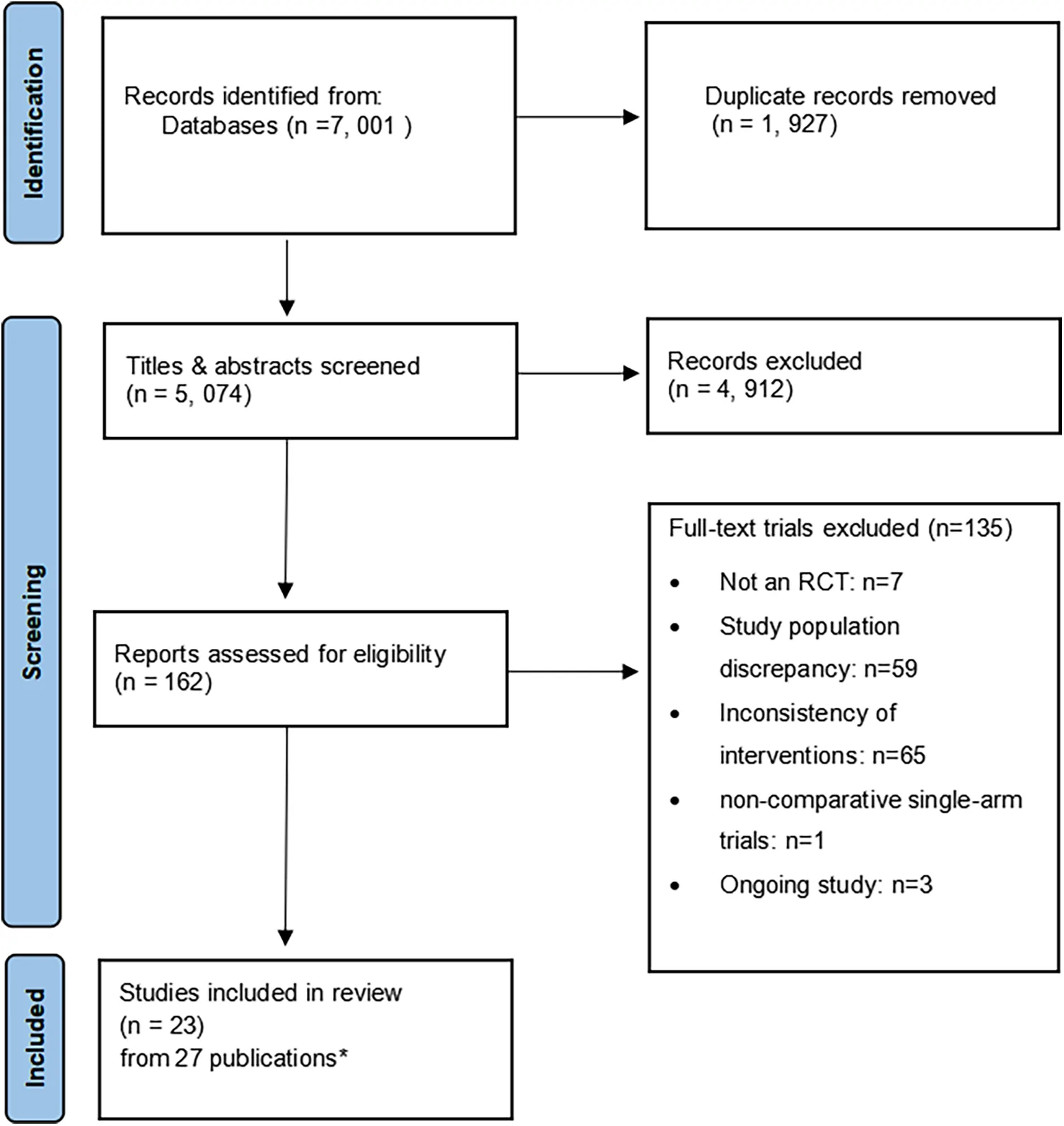
PRISMA flow diagram of study selection.

### 3.2 Characteristics of included studies

The median age of the patients included in this review was 51.7 years, with females comprising 58.6% of the study population. Among the 11 trials (29–31, 33, 34, 37, 39, 41, 51, 52, 54) reporting CEAP clinical classification stage for patients with GSV reflux, the distribution was as follows: 2 trials (37, 51) included patients at C2-C3, 1 trial (33) at C2-C4 stage, 1 trial (31) at C2-C5, 1 trial (39) at C3-C4 stage, 4 trials (29, 30, 34, 41) at C2-C6, 1 trial (52) at C4-C6 stage, 1 trial (54) at C1-C6 stage.

Regarding intraoperative guiding modalities adopted during foam injection and subsequent follow-up assessments across included studies, ultrasound guidance was applied in 13 studies (29–31, 33, 35, 37, 39, 41, 43, 44, 46, 48, 52), x-ray fluoroscopic guidance in 5 studies (34, 40, 47, 53, 54), angiographic guidance in 2 studies (32, 42), and guidance modality remained unreported in another 3 studies (36, 38, 45).

Two trials (31, 34) utilized foam sclerotherapy for repeat treatment of GSV reflux, this retreatment was pre-planned as part of routine therapy.

All surgical interventions across the trials consisted of HL/S. None of the included trials performed concomitant phlebectomy during the intervention procedure. The follow-up duration ranged from 0.5 to 168 months, with an interquartile range (IQR) of 6–42 months (Table 1). In addition, none of the included studies performed concomitant phlebectomy.

**Table 1:** Baseline characteristics of 23 eligible randomized controlled trials.

Study	Countries	Age (Mean ± SD)	Female, %	Number of participants at baseline, *n*	CEAP stages	Groups wearing stretch socks	Foam specs	GSV diameter (Mean ± SD)	The longest follow-up (months)
Rasmussen 2011^a^	Denmark	50.5 ± 9.17	76.6	250	C2–C6	a: 30 mmHg stocking × 2weeks b: 20mmHg stocking × 2weeks	Tessari method 1:4 liquid-to-gas ratio 1% polidocanol	8.3 ± 2.42	12
Shadid 2012^b^	Netherland	51.18 ± 13.4	73.3	460	C2–C5	a: Bandage 48 h; 30 mmHg stocking × 2 weeks b: Bandage 48 h; 20 mmHg stocking × 2 weeks	Tessari method 1:4 liquid-to-gas ratio 2% polidocanol	6.4 ± 2.2	24
Zhang W 2012	China	72.6 ± 5.83	55	80	C2–C3	a: compression stockings	Tessari method 1:4 liquid-to-gas ratio 1% polidocanol	NR	6
Biemans 2013^c^	Netherland	54.1 ± 14.51	67.6	160	C2–C6	a: Bandage 48 h; 30 mmHg stocking × 2 weeks b: Bandage 48 h; 20 mmHg stocking × 2 weeks	Tessari method 1:3 liquid-to-gas ratio 1% polidocanol	6 ± 1.4	12
Brittenden 2015	UK	49.2 ± 13.7	56.5	575	C2–C6	cohesive bandage: 10 days for both groups	Tessari method 1:3 liquid-to-gas ratio STS 3% (trunk)/1% (tributaries)	NR	6
Chen J 2015	China	51.4 ± 10.23	63.8	80	C2–C3	a: walk 30 min post-procedure; switch to compression stockings × 24 h	Tessari method 1:4 liquid-to-gas ratio 1% polidocanol	NR	1
Lai J 2015	China	46.03 ± 6	59.4	106	C2–C6	a: compression stockings	Tessari method 1:4 liquid-to-gas ratio 1% polidocanol	NR	NR
Venermo 2016^d^	Finnish	48.7 ± 12.04	80.1	155	C2–C4	a: 3 days continuous compression, then daytime wear × 11 days b: bandage ×48 h, daytime compression stocking ×12days	Tessari method, 1:4 liquid-to-gas ratio 1%/3% STS, 1% polidocanol	6.5 ± 1.12	1
Ou YS 2017	China	47.7 ± 8.18	40.5	84	NR	Routine compression bandaging for both groups	Tessari method, 1:4 liquid-to-gas ratio 1% polidocanol	NR	NR
Wang T 2018	China	45.2 ± 3.31	45.3	86	NR	a: elastic bandage at injection site; brisk walk 30 min, switch to compression stockings ×24 h	Tessari method 1:4 liquid-to-gas ratio 1% polidocanol	NR	NR
Wu M 2019	China	38.8 ± 8.15	54.8	104	NR	NR	Initial bolus:2 mL; supplementary injection≤1 mL	NR	NR
Wang L 2019	China	54.9 ± 6.1	46.4	84	NR	a: 15 min local compression per injection + compression stockings b: Post-operative elastic bandage	Tessari method 1:4 liquid-to-gas ratio 1% polidocanol	NR	NR
Shan SY 2019	China	56.8 ± 5.08	53.3	90	NR	a: compression stockings b: elastic bandage	Tessari method 1:4 liquid-to-gas ratio 1% polidocanol	NR	NR
Fu XW 2020	China	51.9 ± 3.63	36.5	52	NR	a: 15 min local compression per injection + compression stockings b: Post-operative elastic bandage	Tessari method 1:4 liquid-to-gas ratio 1% polidocanol	NR	NR
Wang AP 2020	China	53.9 ± 8.68	40.3	62	NR	NR	NR	NR	NR
Han YQ 2020	China	56.5 ± 3.48	63.8	80	NR	a: compression stockings ×1 month b: elastic bandage	Tessari method 1:4 liquid-to-gas ratio 1% polidocanol	NR	6
Li J 2020	China	53.6 ± 4.63	46	100	NR	a: bandage + compression stockings b: elastic bandage	Tessari method 1:4 liquid-to-gas ratio 1% polidocanol	NR	0.5
Yang PG 2021	China	45.65 ± 2.65	NR	60	NR	NR	Tessari method, 1:1 liquid-to-gas ratio 1% polidocanol	NR	NR
Yang DH 2022	China	57.9 ± 3.53	57.5	80	NR	a: continuous stocking wear ×72 h; daytime-only stocking use thereafter per clinical condition	Tessari method, 1:4 liquid-to-gas ratio 1% polidocanol	NR	NR
Gong RL 2022	China	58.5 ± 7.46	35	60	C3–C4	Elastic bandage: 5 days for both groups	Tessari method 1:4 liquid-to-gas ratio 1% polidocanol	NR	NR
Wu XH 2026	China	54.4 ± 8.18	58.1	86	C4-C6	compression stockings: 3 months for both groups	Tessari method 1:4 liquid-to-gas ratio 1% polidocanol	NR	3
Zhang YP 2024	China	46.6 ± 5.17	43.9	66	NR	a: compression stockings ×2 month b: elastic bandage ×2weeks	Tessari method 1:4 liquid-to-gas ratio 1% polidocanol	NR	1
Zhou XW 2024	China	54.9 ± 4.73	55.6	90	C1–C6	a: elastic bandage ×48 h, class II compression stockings ×2months b: elastic bandage ×14days, then switch to compression stockings	Tessari method 1:4 liquid-to-gas ratio NR	<8 cm	2

a, Treatment group; b, control group; CEAP, clinical etiology anatomic pathophysiologic; GSV, great saphenous veins; *N* (%), number of participants; NR, not report; SD, standard deviation; STS, sodium tetradecyl sulfate; UK, United Kingdom.

a The trial included Rasmussen 2011 and Lawaetz 2017 at different follow-up time points.

b The trial included Shadid 2012 and Lam 2018 at different follow-up time points.

c The trial included Biemans 2013 and Velden 2015 at different follow-up time points.

d The trial included Venermo 2016 and Rahman 2026 at different follow-up time points.

### 3.3 Risk of bias

Among the 23 eligible trials, all were assessed as having a risk of bias in at least one domain (Supplementary materials eTable 1 and eFigure 1). Sixteen (69.6%) adequately described the method for generating the randomization sequence, the remaining seven (30.4%) mentioned random allocation without specifying the exact method and were classified as unclear risk for this domain. Allocation concealment was appropriately implemented in five trials (21.7%), the remaining 18 trials did not report allocation concealment methods and were classified as unclear risk. Regarding blinding, three trials (13.0%) explicitly mentioned that blinding was not used, and one (4.3%) reported blinding of outcome assessors, the remaining 19 trials (82.6%) did not mention whether blinding was performed. However, it is generally recognized that blinding of surgeons and patients is not technically feasible in the context of HL/S. one trial (34) were at high risk of bias due to missing outcome data exceeding 20%, with attrition rates of 28.1%. All 23 included trials were assessed as free of selective reporting (Supplementary materials eTable 1, eFigure 1).

### 3.4 Outcomes for foam sclerotherapy vs. HL/S

#### 3.4.1 Primary outcome

Low certainty evidence from 9 RCTs (37–42, 46, 48, 52) and 736 patients suggests foam sclerotherapy may improve symptom relief compared with HL/S (RR, 1.09, 95% CI, 1.03 to 1.16; ARR, 7.7%, 95% CI, 2.6% to 13.7%; Supplementary materials eFigure 2). Low certainty evidence from 6 RCTs (30, 32, 34–36, 51) and 1,164 patients suggests foam sclerotherapy may be associated with lower complete GSV occlusion rates compared with HL/S. (RR 0.65, 95% CI, 0.49 to 0.86; ARR, 28%, 95% CI, 11.2% to 40.8%; Figure 2). Very low certainty evidence suggested that there was no statistically significant difference in recurrence rate between foam sclerotherapy and HL/S (8 RCTs (10, 35, 36, 40, 43, 45, 50, 54) and 966 patients (RR, 0.56, 95% CI, 0.25 to 1.23; ARR, 9.9%, 95% CI, −5.2% to 16.8%; Figure 3) (Table 2).

**Figure 2:**
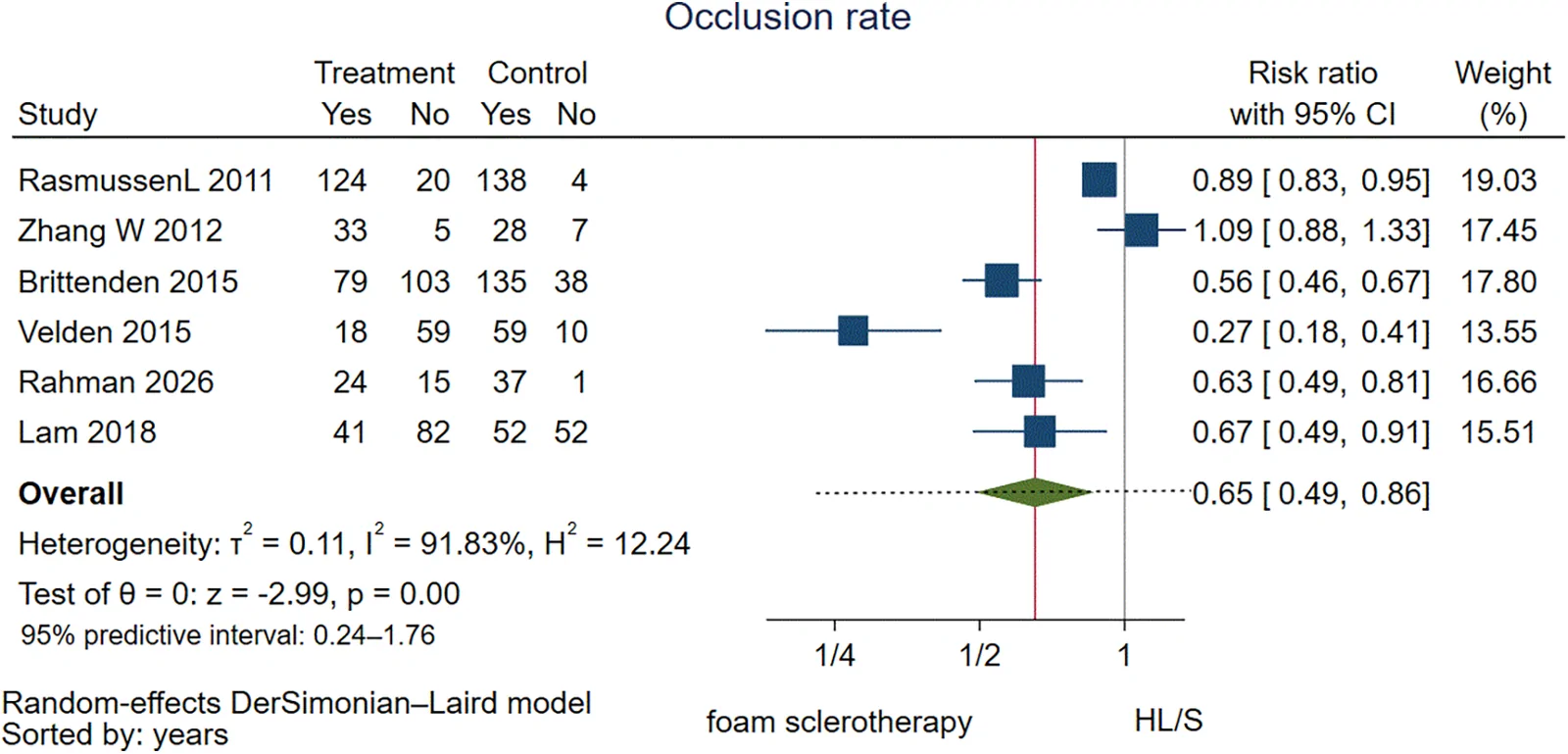
Forest plot for occlusion rate of GSV reflux: UGFS versus HL/S.

**Figure 3:**
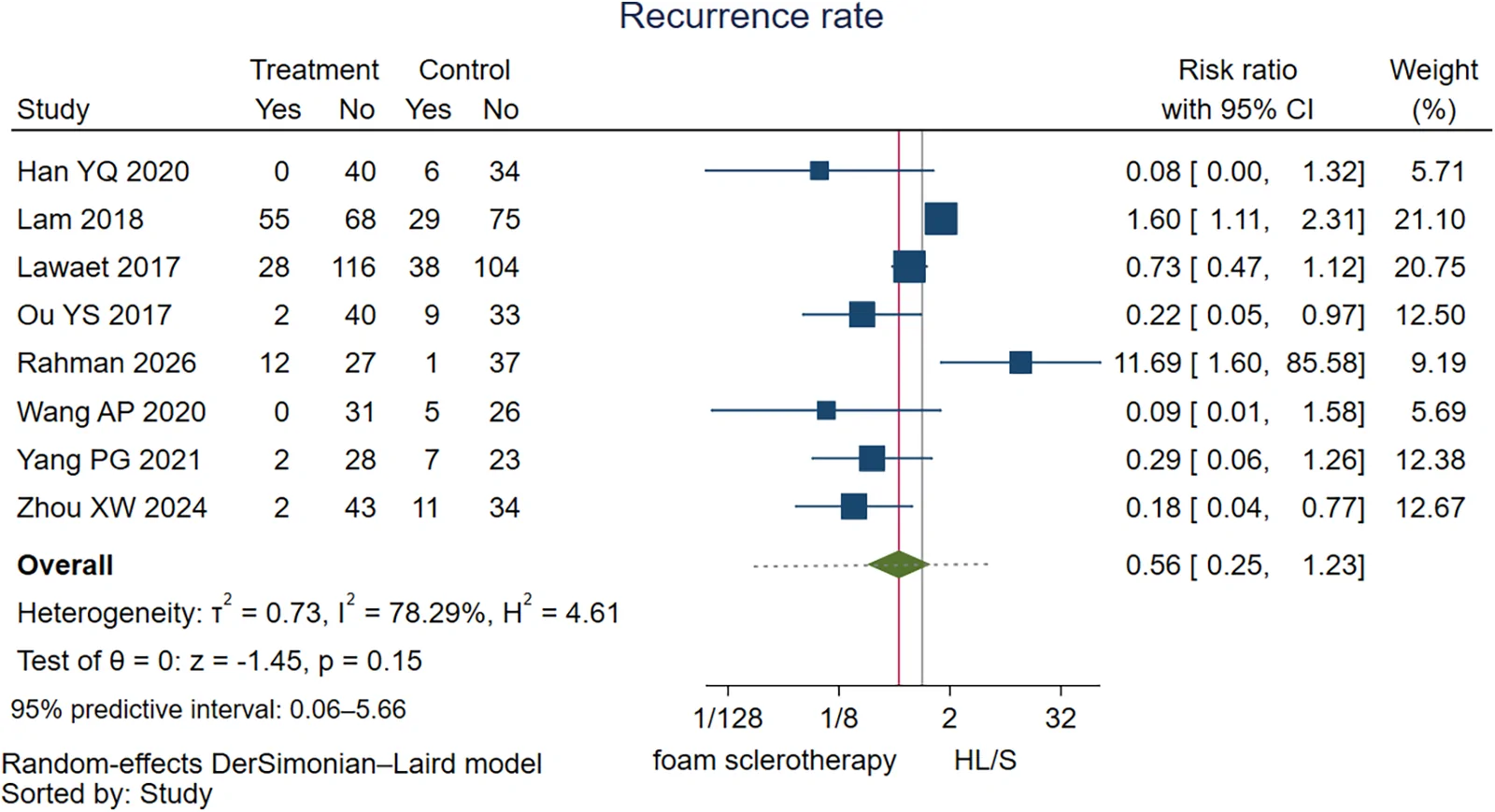
Forest plot for recurrence rate of GSV reflux: UGFS versus HL/S.

**Table 2:** GRADE evidence profile for occlusion rate, recurrence rate, symptom improvement in with GSV reflux treated with foam sclerotherapy.

Outcome	No. of trials (No. of patients)	Risk of bias	Inconsistency	Indirectness	Imprecision	Publication bias	Treatment association (95% CI)		Overall certainty of evidence
							RR	ARR	
Occlusion rate	6 (1164)	Yes	I^2^ = 91.83%	NO	NO	NA	0.65 (0.49 to 0.86)	28% (11.2% to 40.8%)	Low^a^
Recurrence rate	8 (966)	Yes	I^2^ = 78.29%	NO	Yes	NA	0.56 (0.25 to 1.23)	9.9% (−5.2% to 16.8%)	Very low^b^
Symptom improvement	9 (736)	Yes	I^2^ = 68.64%	NO	NO	NA	1.09 (1.03 to 1.16)	7.7% (2.6% to 13.7%)	Low^a^

ARR, Absolute Risk Reduction; CI, confidence interval; GSV, great saphenous veins; NA, not applicable due to less than 10 studies; RR, relative risk.

a Downgraded 2 level due to risk of bias and inconsistency.

b Downgraded 3 level due to risk of bias, inconsistency, and imprecision.

#### 3.4.2 Secondary outcome indicators

Low to very low certainty evidence suggests foam sclerotherapy may be associated with shorter surgery duration [15 RCT (30, 34, 37, 38, 40, 41, 43–47, 49, 50, 53, 54) and 1,843 patients, WMD −35.28 min, 95% CI −45.80 to −24.75 min; Supplementary materials eFigure 3], a reduced length of hospital stay (12 RCTs (37, 38, 40, 41, 43–47, 49, 50, 54) and 954 patients, WMD −3.72 days, 95% CI −4.76 to −2.68 days; Supplementary materials eFigure 4), lower hospital costs [6 RCTs (30, 31, 37, 38, 44, 47) and 1,263 patients, WMD −4,622.34 €, 95% CI −6,340.55 to −2,904.14 €; Supplementary materials eFigure 5] and less volume of blood loss [9RCTs (37, 43, 45–47, 49, 50, 53, 54) and 692 patients, WMD −50.48 mL, 95% CI −60.34 to −40.61 mL; Supplementary materials eFigure 6] compared with HL/S (Table 3).

**Table 3:** GRADE evidence profile for duration of surgery, hospital stay, hospital costs and volume of blood loss in patients with GSV reflux treated with foam sclerotherapy.

Outcome	No of trials (No of patients)	Risk of bias	Inconsistency	Indirectness	Imprecision	Publication bias	Treatment association (95% CI)	Overall certainty of evidence
Duration of surgery (min)	15 (1, 843)	Yes	I^2^ = 99.18%	NO	NO	detected; Symmetric funnel plot; egger's test *P* < 0.001	WMD −35.28min (95% CI −45.80 to −24.75)	Very low^b^
Hospital stay (day)	12 (954)	Yes	I^2^ = 97.16%	NO	NO	detected; Symmetric funnel plot; egger's test *P* < 0.001	WMD −3.72day (95% CI −4.76 to −2.68)	Very low^b^
Hospital costs (€)	6 (1,263)	Yes	I^2^ = 98.59%	NO	NO	NA	WMD −4,622.34€ (95% CI −6,340.55 to −2,904.14）	Low^a^
Volume of blood loss (mL)	9 (692)	Yes	I^2^ = 99.32%	NO	NO	NA	WMD −50.48mL (95% CI −60.34 to −40.61)	Low^a^

CI, confidence interval; GSV, great saphenous veins; NA, not applicable due to less than 10 studies; RR, relative risk; WMD, weighted mean difference.

a Downgraded 2 levels due to risk of bias and inconsistency.

b Downgraded 3 levels due to risk of bias, inconsistency, and risk of publication bias.

#### 3.4.3 Other outcome indicators

##### 3.4.3.1 Venous clinical severity score (VCSS)

Low certainty evidence from 3 RCTs (30, 35, 42) (1,105 patients) may find no significant difference in the VCSS between foam sclerotherapy and HL/S group. (WMD −0.20 points, 95% CI −0.45 to 0.05 points on the 30-point VCSS; Supplementary materials eFigure 7 and eTable 2).

##### 3.4.3.2 Quality of life

Low certainty evidence from 2 RCTs (30, 34) (723 patients) found that foam sclerotherapy had no significant effect on the Physical Functioning domain of the quality of life compared to HL/S (WMD −0.50 points, 95% CI −1.93 to 0.93 on a 100-point scale Supplementary materials eFigure 8 and eTable 2).

##### 3.4.3.3 Patient satisfaction

Only 2 of the 23 included trials RCTs (31, 39) (418 patients) reported on patient satisfaction. Very low-certainty evidence suggested that patient satisfaction may be higher in the foam sclerotherapy group than in the HL/S group, although the result was not statistically significant (RR 1.12, 95% CI 0.84 to 1.48; ARR 10.2%, 95% CI −13.6% to 40.8%; Supplementary materials eFigure 9 and eTable 3).

##### 3.4.3.4 Safety analysis

Low certainty evidence indicated that, compared with HL/S, the foam sclerotherapy group was associated with a lower risk of several postoperative complications, including infections [8 RCTs (29, 31, 33, 34, 40, 45, 48, 52) and 1,283 patients, RR: 0.28, 95% CI: 0.10 to 0.76; ARR: 2.55%, 95% CI: 0.85% to 3.19%; Supplementary materials eFigure 10], numbness [7 RCTs (30, 37, 38, 46, 47, 53, 54) and 945 patients, RR: 0.25, 95% CI: 0.14 to 0.42; ARR: 10%, 95% CI: 7.73% to 11.47%; Supplementary materials eFigure 11], and haematoma [6 RCTs (31, 33, 40, 48, 50, 52) and 893 patients, RR: 0.27, 95% CI: 0.16 to 0.44; ARR: 8.9%, 95% CI: 6.84% to 10.25%; Supplementary materials eFigure 12]. Low to very low certainty evidence found no significant differences may exist between the two groups for others adverse effects, such as pulmonary embolism, paraesthesia, phlebothrombosis, thrombophlebitis, skin ulceration, lumpiness, subcutaneous stasis, hyperpigmentation, and nerve injury (Supplementary materials eFigure 13–21 and eTable 4).

### 3.5 Subgroup analysis and sensitivity analyses

The certainty of most subgroup analyses was limited. Subgroup analysis based on the language of publication (English vs. Non-English) revealed statistically significant differences in several outcome measures, including recurrence rate, duration of surgery, and the incidence of specific adverse events such as hyperpigmentation, and lumpiness (Supplementary materials eFigure 22–30, and eTable 5–7). Similarly, a subgroup analysis evaluating the use of compression stockings also showed significant effects on hyperpigmentation and phlebothrombosis (Supplementary materials eFigure 31–32, and eTable 8). However, the certainty of all these observed subgroup effects was assessed as very low (Supplementary materials eTable 9). We further performed subgroup analyses stratified by follow-up duration (≤5 years vs. >5 years) for occlusion rate and recurrence rate (Supplementary materials eTable 9). For occlusion rate, HL/S yielded superior vessel occlusion outcomes relative to foam sclerotherapy in both short-term (follow-up ≤5 years) and long-term (follow-up >5 years) subgroups; the advantage of HL/S was consistent across different follow-up durations, with non-significant subgroup interaction (*p* = 0.09). For recurrence rate, pooled results of both subgroups and the overall analysis showed no statistically significant difference in recurrence risk between foam sclerotherapy and HL/S; subgroup interaction also did not reach statistical significance (*p* = 0.15), indicating follow-up duration could not produce a statistically significant modification on the relative recurrence risk of the two interventions, despite observed numerical trend of lower recurrence for foam sclerotherapy within 5 years and higher recurrence after 5 years of follow-up.

Sensitivity analysis demonstrated that the number of treatments (single vs. repeated) did not significantly affect the occlusion rate of the GSV. However, repeated treatment was associated with an increased risk of lower extremity infection and hyperpigmentation. (Supplementary materials eTable 11). Furthermore, after excluding two trials (37, 41) with complete symptom remission across both groups for sensitivity analysis, the heterogeneity of the symptom improvement outcome decreased, while the direction of pooled RR remained unchanged (RR: 1.16, 95% CI: 1.07 to 1.25). The results of our pooling analysis using the Hartung-Knapp-Sidik- Jonkman method were consistent with the Der-Simonan-Laird method (Supplementary materials eTable 11–12).

## 4 Discussion

### 4.1 Main findings

This meta-analysis demonstrated that foam sclerotherapy exhibits a distinct profile of advantages and limitations compared with HL/S for the treatment of GSV.

Our primary outcomes suggest foam sclerotherapy may be linked to lower complete GSV occlusion rates relative to HL/S (supported by low-certainty evidence). This finding should be interpreted with caution, as substantial heterogeneity was observed across studies, indicating that the occlusive effect of foam sclerotherapy varied markedly across trials. A possible explanation for the lower occlusion rate is that foam sclerotherapy relies on uniform foam-endothelium contact (55). In larger-diameter trunks, intraluminal blood flow may dilute the foam and prevent complete wall apposition, leading to partial closure and subsequent recanalization (56). Consistent with this mechanism, current guidelines (56) recommend that foam sclerotherapy be used primarily for smaller-diameter GSV to achieve better efficacy. Unfortunately, most studies in this review did not report the diameter of the treated GSV, precluding any analysis of whether the observed lower occlusion rate was associated with vein diameter. Future research should strengthen the reporting of this key variable.

Furthermore, our sensitivity analysis regarding repeated treatment showed that repeated foam sclerotherapy sessions did not significantly improve the occlusion rate, although this finding may be limited by the small number of included studies that used repeated foam sclerotherapy (only two trials) and therefore requires confirmation in future research.

Despite its lower occlusion rate, low-certainty evidence shows that foam sclerotherapy demonstrated advantages over conventional HL/S in symptom improvement. This finding suggests that clinical symptom relief does not depend entirely on complete occlusion of the venous lumen. Relevant guidelines (57, 58) point out that duplex ultrasound-detected venous anatomical occlusion status and symptomatic improvement are two independent, weakly correlated evaluation endpoints for chronic venous disease, which should not be treated as contradictory results. This aligns with the dissociation phenomenon observed in our study, namely the lower anatomical occlusion rates but better symptomatic improvement achieved by foam sclerotherapy. A number of clinical studies (59, 60) have also confirmed that partial recanalization of the GSV after foam sclerotherapy does not inevitably lead to recurrence of venous-related symptoms, and complete trunk occlusion cannot guarantee the complete elimination of lower limb discomfort.

Therefore, for patients with GSV whose primary treatment goal is symptom improvement, especially those who cannot tolerate or are unwilling to undergo open HL/S, foam sclerotherapy may be considered a preferred treatment option**.**

In contrast, no statistically significant difference in recurrence rates was observed between foam sclerotherapy and HL/S. Subgroup analyses stratified by follow-up duration likewise revealed no significant between-group differences in recurrence rates. This finding requires cautious interpretation, as the limited available data reported across included studies may have influenced the comparative results of long-term efficacy.

For secondary outcomes, low to very low certainty evidence suggested benefits of foam sclerotherapy over HL/S in several perioperative metrics, including shorter surgery duration, reduced hospital stay, lower costs, and less blood loss. Therefore, these findings should be interpreted with caution and warrant validation in future high-quality studies.

### 4.2 Comparison with international venous guidelines and previous meta-analyses

The 2022 ESVS and 2023 SVS/AVF/AVLS guidelines have established a hierarchical treatment algorithm for GSV reflux (57, 58). Endovenous thermal ablation (EVTA) is recommended as the first-line therapy for GSV reflux, while non-thermal non-tumescent techniques—cyanoacrylate closure (CAC, Class IIa) and mechanochemical ablation (MOCA, Class IIb)—are recommended as alternative treatment options for appropriately selected patients who prefer non-thermal approaches or have contraindications to tumescent anaesthesia. Foam sclerotherapy, by contrast, is positioned as a third-line option, primarily for smaller-diameter trunks or as an adjunct for residual branch varicosities. Our meta-analysis, however, only compared foam sclerotherapy with HL/S; the absence of head-to-head trials against MOCA or CAC means we cannot directly position foam sclerotherapy relative to these contemporary alternatives.

When compared with HL/S, our findings are largely consistent with seven previous meta-analyses (11–17) regarding symptom improvement and occlusion rate, though discrepancies emerged in recurrence rate. Hamann et al. (17) and van den Bos et al. (14) used “anatomical success rate” as their outcome measure—defined as complete occlusion or absence of reflux—which is essentially the same as our occlusion rate definition. Hamann et al. reported that foam sclerotherapy was associated with a lower anatomical success rate than HL/S, aligning with our results. However, van den Bos et al., whose analysis included both RCTs and case series, found foam sclerotherapy comparable to HL/S in anatomical success—a difference likely attributable to study design heterogeneity. Regarding recurrence, our analysis found no statistically significant difference between foam sclerotherapy and HL/S. This contrasts with Hamann et al., who reported a higher long-term recurrence rate with foam sclerotherapy, but aligns with Kheirelseid et al. (13) and Whing et al. (11), who similarly found no significant difference in either short- or long-term follow-up. These inconsistencies likely reflect variability in recurrence definitions, follow-up durations, and the quantity of RCT data available.

Whing et al. defined technical success as post-treatment venous occlusion without reflux—consistent with our occlusion rate definition—and demonstrated that HL/S had a clinical advantage over foam sclerotherapy within 1 to 3 years (low certainty) and that foam sclerotherapy's technical success was significantly lower than HL/S beyond 5 years (moderate certainty), both consistent with our findings.

### 4.3 Strengths and limitations

This systematic review has several methodological strengths that support the validity of the findings. First, it incorporates a larger number of RCTs, which represent the highest level of evidence in interventional studies. Second, we used the GRADE approach to appraise the certainty of evidence and converted all significant pooled mean effects to ARRs to facilitate clinical interpretation. Third, we conducted predefined subgroup analyses to explore potential sources of heterogeneity and formally assessed the certainty of all potential subgroup effects. In addition, this review provides pooled estimates for several important perioperative outcomes, including hospital stay, hospital costs, operative duration, blood loss, and safety profiles.

Several limitations should also be acknowledged. First, the composite endpoint of “symptom improvement” was derived from heterogeneous, non-validated definitions across studies, and recurrence was also defined inconsistently; pooling such diverse measures may introduce measurement bias. Second, key prognostic variables—including follow-up duration, CEAP clinical classification stage, and GSV diameter—were insufficiently reported in a stratified manner in most trials (55, 56). These factors are well-established strong determinants of treatment success and recurrence; their incomplete reporting precluded adjustment for potential confounding and limited our ability to perform planned subgroup analyses. This likely contributed to the substantial heterogeneity observed across studies, particularly for perioperative outcomes and occlusion rate, where I² exceeded 90%. Additional sources of heterogeneity included inconsistent foam preparation protocols, variable sclerosant types and concentrations, and unmeasured operator experience—factors that were rarely documented in the included trials. Third, all included RCTs used conventional HL/S as the comparator rather than endovenous thermal ablation—the current first-line therapy—which limits the applicability of our findings to contemporary clinical practice. Fourth, the geographic distribution of the included trials was heavily skewed: 18 of the 23 RCTs were single-center studies conducted in China with small sample sizes and inadequate reporting of allocation concealment and blinding, whereas only five high-quality Western multicenter trials adopted rigorous risk-of-bias control. This geographic clustering, together with regional variations in disease management standards, sclerosant protocols, and reimbursement systems, may introduce bias and reduce the global generalizability of our pooled estimates. Fifth, none of the included trials employed standardized patient-reported outcome measures, and cost data were converted to euros without inflation adjustment across studies spanning more than a decade; neither limitation was addressed in the original studies, making cross-trial comparisons of these secondary outcomes less reliable.

## 5 Conclusion

This systematic review and meta-analysis demonstrate that compared with HL/S, foam sclerotherapy provides multiple perioperative benefits but yields inferior anatomical occlusion rates, with low overall certainty of evidence. No significant difference in recurrence rates was observed, but this should not be interpreted as evidence of comparable long-term efficacy, given inconsistent recurrence definitions, wide variation in follow-up periods, and divergent recurrence mechanisms. The substantial heterogeneity across studies further limits the robustness of pooled estimates. Future high-quality trials with standardized outcome definitions, consistent reporting of key prognostic variables (e.g., GSV diameter, CEAP stage), and longer follow-up are warranted to strengthen the evidence base.

## Data Availability

The original contributions presented in the study are included in the article/Supplementary Material, further inquiries can be directed to the corresponding authors.
