# Supplementary material for: Efficacy and safety of foam sclerotherapy for great saphenous vein reflux: a systematic review and meta-analysis of RCTs
Source: Front Med (Lausanne). 2026 Sep 14;13:1903287. doi: 10.3389/fmed.2026.1903287 (PMC13616788; doi:10.3389/fmed.2026.1903287)
Supplement: Supplementary file 1 [file Datasheet1.docx]

eAppendix

contents

[eTable 1 Risk of bias assessment of 23 eligible randomized controlled trials	3](#_Toc18949)

[eTable 2 GRADE evidence for quality of life in patients with GSV reflux treated with foam sclerotherapy.	4](#_Toc9654)

[eTable 3 GRADE evidence for Satisfaction rate in patients with GSV reflux treated with foam sclerotherapy.	4](#_Toc32237)

[eTable 4 GRADE evidence for adverse events in patients with GSV reflux treated with foam sclerotherapy.	5](#_Toc28759)

[eTable 5 Subgroup analysis of occlusion rate, recurrence rate, and clinical symptom improvement in patients treated with foam sclerotherapy vs.HL/S.	7](#_Toc12568)

[eTable 6 Subgroup analysis of duration of surgery, hospital costs, volume of blood loss and hospital stay in patients treated with foam sclerotherapy vs. HL/S.	8](#_Toc19274)

[eTable 7 Subgroup analysis of adverse events in GSV reflux patients treated with foam sclerotherapy vs.HL/S: Non-English or English	9](#_Toc14852)

[eTable 8 Subgroup analysis of adverse events in GSV reflux patients treated with foam sclerotherapy vs. HL/S with or without compression stockings	10](#_Toc32210)

[eTable 9 Subgroup analysis of occlusion rate and recurrence rate among patients with GSV refux treated with foam sclerotherapy vs. HL/S stratified by follow-up duration.	11](#_Toc27301)

[eTable 10 ICEMAN criteria or assessing the credibility of subgroup effects	12](#_Toc746)

[eTable 11 Sensitivity analysis for binary outcomes	15](#_Toc18320)

[eTable 12 Sensitivity analysis for continuous outcomes	17](#_Toc5168)

[eFig. 1 Risk of bias assessment of 23 eligible randomized controlled trials	18](#_Toc21956)

[eFig. 2 Symptom improvement of GSV reflux treated with foam sclerotherapy vs. HL/S.	19](#_Toc29982)

[eFig. 3 Duration of surgery after foam sclerotherapy vs. HL/S treatment of GSV reflux.	19](#_Toc7041)

[eFig. 4 Hospital stay after foam sclerotherapy vs. HL/S treatment of GSV reflux.	20](#_Toc18672)

[eFig. 5 Hospital costs after foam sclerotherapy vs. HL/S treatment of GSV reflux.	20](#_Toc5387)

[eFig. 6 Volume of blood loss after foam sclerotherapy vs. HL/S treatment of GSV reflux.	21](#_Toc20722)

[eFig. 7 VCSS score after foam sclerotherapy vs. HL/S treatment of GSV reflux.	21](#_Toc9290)

[eFig. 8 Quality of life after foam sclerotherapy vs. HL/S treatment of GSV reflux.	22](#_Toc7689)

[eFig. 9 Patient satisfaction after foam sclerotherapy vs. HL/S treatment of GSV reflux.	22](#_Toc31398)

[eFig. 10 Incidence of infection after foam sclerotherapy vs. HL/S treatment of GSV reflux.	23](#_Toc22449)

[eFig. 11 Incidence of numbness after foam sclerotherapy vs. HL/S treatment of GSV reflux.	23](#_Toc13973)

[eFig. 12 Incidence of haematoma after foam sclerotherapy vs. HL/S treatment of GSV reflux.	24](#_Toc3021)

[eFig. 13 Incidence of pulmonary embolism after foam sclerotherapy vs. HL/S treatment of GSV reflux.	24](#_Toc31402)

[eFig. 14 Incidence of paraesthesia after foam sclerotherapy vs. HL/S treatment of GSV reflux.	25](#_Toc22001)

[eFig. 15 Incidence of phlebothrombosis after foam sclerotherapy vs.HL/S treatment of GSV reflux.	25](#_Toc12853)

[eFig. 16 Incidence of thrombophlebitis after foam sclerotherapy vs. HL/S treatment of GSV reflux.	26](#_Toc25107)

[eFig. 17 Incidence of skin ulceration after foam sclerotherapy vs. HL/S treatment of GSV reflux.	26](#_Toc6055)

[eFig. 18 Incidence of lumpiness after foam sclerotherapy vs. HL/S treatment of GSV reflux.	27](#_Toc11727)

[eFig. 19 Incidence of subcutaneous stasis after foam sclerotherapy vs. HL/S treatment of GSV reflux.	28](#_Toc29532)

[eFig. 20 Incidence of hyperpigmentation after foam sclerotherapy vs. HL/S treatment of GSV reflux.	29](#_Toc17557)

[eFig. 21 Incidence of nerve injury after foam sclerotherapy vs.HL/S treatment of GSV reflux.	29](#_Toc32125)

[eFig. 22 Subgroup Analysis: Comparison of Recurrence Rates Between foam sclerotherapy and HL/S for GSV reflux in Non-English versus English Publications	30](#_Toc22140)

[eFig. 23 Subgroup Analysis: Comparison of Duration of surgery Between foam sclerotherapy and HL/S for GSV reflux in Non-English versus English Publications	31](#_Toc25614)

[eFig. 24 Subgroup Analysis: Comparison of Hospital Costs Between foam sclerotherapy and HL/S for GSV reflux in Non-English versus English Publications	32](#_Toc18071)

[eFig. 25 Subgroup Analysis: Comparison of Adverse Event Pulmonary Embolism Between foam sclerotherapy and HL/S for GSV reflux in Non-English versus English Publications	33](#_Toc15984)

[eFig. 26 Subgroup Analysis: Comparison of Adverse Event Infection Between foam sclerotherapy and HL/S for GSV reflux in Non-English versus English Publications	34](#_Toc29945)

[eFig. 27 Subgroup Analysis: Comparison of Adverse Event Phlebophlogosis Between foam sclerotherapy and HL/S for GSV reflux in Non-English versus English Publications	35](#_Toc16853)

[eFig. 28 Subgroup Analysis: Comparison of Adverse Event Lumpiness Between foam sclerotherapy and HL/S for GSV reflux in Non-English versus English Publications	36](#_Toc27082)

[eFig. 29 Subgroup Analysis: Comparison of Adverse Event Hyperpigmentation Between foam sclerotherapy and HL/S for GSV reflux in Non-English versus English Publications	37](#_Toc26238)

[eFig. 30 Subgroup Analysis: Comparison of Adverse Event Hematoma Between foam sclerotherapy and HL/S for GSV reflux in Non-English versus English Publications	38](#_Toc29214)

[eFig. 31 Subgroup Analysis: Comparison of Adverse Event Thrombophlebitis Between foam sclerotherapy and HL/S for GSV reflux in Treatment Group Wore Socks versus Both Groups Wore Socks.	39](#_Toc27575)

[eFig. 32 Subgroup Analysis: Comparison of Adverse Event Hyperpigmentation Between foam sclerotherapy and HL/S for GSV reflux in Treatment Group Wore Socks versus Both Groups Wore Socks.	40](#_Toc1135)

[eFig. 33 Subgroup Analysis: Comparison of Occlusion Rates Between foam sclerotherapy and HL/S for GSV reflux in Follow up ≤ 5 years versus Follow up ＞ 5 years	41](#_Toc22767)

[eFig. 34 Subgroup Analysis: Comparison of Recurrence Rates Between foam sclerotherapy and HL/S for GSV reflux in Follow up ≤ 5 years versus Follow up ＞ 5 years	42](#_Toc12769)

[Search strategies	43](#_Toc19557)

# eTable 1 Risk of bias assessment of 23 eligible randomized controlled trials

| **study** | Adequate randomization sequence generation | Adequate allocation concealment | Blinding of outcome assessors | Loss to follow-up(%) | Selective report |
| --- | --- | --- | --- | --- | --- |
| Rasmussen 2011 | Yes | Yes | No | 28.1 | NO^#^ |
| Shadid 2012 | Yes | Yes | Yes | 9.3 | NO |
| Zhang W 2012 | Unclear† | Unclear† | Unclear | 0 | NO^#^ |
| Biemans 2013 | Yes | Yes | No | 0.6 | NO |
| Chen J 2015 | Unclear† | Unclear† | Unclear | 0 | NO^#^ |
| Brittenden 2015 | Yes | Yes | No | 0 | NO |
| Lai J 2015 | Unclear† | Unclear† | Unclear | 0 | NO^#^ |
| Venermo 2016 | Yes | Yes | No | 5.5 | NO^#^ |
| Ou YS 2017 | Unclear† | Unclear† | Unclear | 0 | NO^#^ |
| Wang T 2018 | Yes | Unclear† | Unclear | 0 | No^#^ |
| Wu M 2019 | Yes | Unclear† | Unclear | 0 | NO^#^ |
| Wang L 2019 | Yes | Unclear† | Unclear | 0 | NO^#^ |
| Shan SY 2019 | Yes | Unclear† | Unclear | 0 | NO^#^ |
| Li J 2020 | Yes | Unclear† | Unclear | 0 | NO^#^ |
| Fu XW 2020 | Unclear† | Unclear† | Unclear | 0 | NO^#^ |
| Wang AP 2020 | Yes | Unclear† | Unclear | 0 | NO^#^ |
| Han YQ 2020 | Yes | Unclear† | Unclear | 0 | NO^#^ |
| Yang PG 2021 | Yes | Unclear† | Unclear | 0 | NO^#^ |
| Yang DH 2022 | Yes | Unclear† | Unclear | 0 | NO^#^ |
| Gong RL 2022 | Unclear† | Unclear† | Unclear | 0 | NO^#^ |
| Zhang YP 2024 | Yes | Unclear† | Unclear | 0 | NO^#^ |
| Zhou XW 2024 | Yes | Unclear† | Unclear | 0 | NO^#^ |
| WU XH 2026 | Yes | Unclear† | Unclear | 0 | NO^#^ |

†: Study that did not report random sequence generation or allocation concealment measures were rated as having unclear risk of bias.

#: The registration number of procotol was not reported, and only the outcome indicator reported in the methods section corresponded to the outcome indicator in the results.

# eTable 2 GRADE evidence for quality of life and VCSS in patients with GSV reflux treated with foam sclerotherapy.

| **Outcome** | **No of trials**  **(No of patients)** | **Risk of bias** | **Inconsistency** | **Indirectness** | **Imprecision** | **Publication bias** | **Treatment association (95% CI)** | **Overall quality of evidence** |
| --- | --- | --- | --- | --- | --- | --- | --- | --- |
| Quality of Life | 2 (723) | Yes | I^2^ = 0.00% | NO | Yes | NA | WMD -0.50  (95%CI -1.93 to 0.93) | Low† |
| VCSS | 3 (1105) | Yes | I^2^ = 0.00% | NO | Yes | NA | WMD −0.20  (95%CI −0.45 to 0.05) | Low† |

**WMD:** weighted mean difference; **CI:** confidence interval; **NA:** not applicable due to less than 10 studies.

†: Downgraded 2 levels due to risk of bias and imprecision.

# eTable 3 GRADE evidence for Satisfaction rate in patients with GSV reflux treated with foam sclerotherapy.

| **Outcome** | **No of trials**  **(No of patients)** | **Risk of bias** | **Inconsistency** | **Indirectness** | **Imprecision** | **Publication bias** | **Treatment association (95% CI)** | | **Overall quality of evidence** |
| --- | --- | --- | --- | --- | --- | --- | --- | --- | --- |
|  |  |  |  |  |  |  | RR | Absolute Risk Reduction |  |
| Satisfaction rate | 2 (418) | Yes | I^2^ = 82.79% | NO | Yes | NA | 1.12 (0.84 to 1.48) | 10.2% (-13.6% to 40.8%) | Very low^§^ |

**RR:** relative risk; **CI:** 95% confidence interval; **NA:** not available to conduct publication bias assessment for less than 10 studies in a given.

§: Downgraded 3 levels due to risk of bias, inconsistency and imprecision.

# eTable 4 GRADE evidence for adverse events in patients with GSV reflux treated with foam sclerotherapy.

| **Outcome** | **No of trials**  **(No of patients)** | **Risk of bias** | **Inconsistency** | **Indirectness** | **Imprecision** | **Publication bias** | **Treatment association (95% CI)** | | **Overall quality of evidence** |
| --- | --- | --- | --- | --- | --- | --- | --- | --- | --- |
|  |  |  |  |  |  |  | RR | Absolute Risk Reduction |  |
| Haematoma | 6 (893) | YES | I^2^ = 0% | NO | YES | NA | 0.27 (0.16 to 0.44) | 8.9% (6.84% to 10.25%) | Low* |
| Nerve injury | 2 (120) | YES | I^2^ = 0% | NO | YES | NA | 0.17 (0.02 to 1.36) | 6.92% (-3% to 8.17%) | Very low^‡^ |
| Subcutaneous stasis | 7 (969) | YES | I^2^ = 12.10% | NO | YES | NA | 0.61 (0.33 to 1.13) | 4.82% (-1.61% to 8.29%) | Very low^‡^ |
| Thrombophlebitis | 10 (1267) | YES | I^2^ = 52.26% | NO | YES | detected;  Asymmetric funnel plot; Harbord’s test P=0.004 | 0.71 (0.26 to 1.92) | 1.22% (-3.88% to 3.13%) | Very low^&^ |
| Infection | 8 (1283) | YES | I^2^ = 2.21% | NO | YES | NA | 0.28 (0.10 to 0.76) | 2.55% (0.85% to 3.19%) | Low* |
| Pulmonary embolism | 5 (980) | YES | I^2^ = 0% | NO | YES | NA | 0.78 (0.21 to 2.95) | -0.19% (-1.67% to 0.68%) | Very low^‡^ |
| Lumpiness | 7 (1010) | YES | I^2^ = 76.04% | NO | YES | NA | 0.93 (0.42 to 2.02) | 1.01% (-14.72% to 8.37%) | Very low^§^ |
| Numbness | 7 (945) | YES | I^2^ = 0% | NO | YES | NA | 0.25 (0.14 to 0.42) | 10.0%(7.73% to 11.47%) | Low^*^ |
| Skin ulceration | 2 (567) | YES | I^2^ = 52.43% | NO | YES | NA | 0.96 (0.04 to 21.27) | 0.29%(-14.69% to 0.7%) | Very low^‡^ |
| Phlebothrombosis | 4 (898) | YES | I^2^ = 0% | NO | YES | NA | 0.76 (0.25 to 2.34) | 0.34%(-1.88% to 1.05%) | Very low^‡^ |
| Paraesthesia | 4 (951) | YES | I^2^ = 0% | NO | YES | NA | 0.43 (0.15 to 1.24) | 1.78%(-0.75% to 2.66%) | Very low^‡^ |
| Hyperpigmentation | 11 (1920) | YES | I^2^ = 66.52% | NO | YES | detected;  Asymmetric funnel plot; Harbord’s test P=0.0006 | 1.51 (0.70 to 3.28) | -2.85%(-12.75% to 1.68%) | Very low^&^ |

**RR:** relative risk; **CI:** 95% confidence interval; **NA:** not available to conduct publication bias assessment for less than 10 studies in a given.

*: Downgraded 2 levels due to risk of bias and imprecision.

‡: Downgraded 3 levels due to risk of bias and imprecision.

§: Downgraded 4 levels due to risk of bias, inconsistency, and imprecision.

&: Downgraded 4 levels due to risk of bias, imprecision, and risk of publication bias.

# eTable 5 Subgroup analysis of occlusion rate, recurrence rate, and clinical symptom improvement in patients treated with foam sclerotherapy vs.HL/S.

| Outcome | Non-English | | | | English | | | | **Test of interaction p-value** |
| --- | --- | --- | --- | --- | --- | --- | --- | --- | --- |
|  | No.  studies | RR | 95%CI | | No.  studies | RR | 95%CI | |  |
| Occlusion rate  (6 studies） | 1 | — | — | — | 5 | — | — | — | NA |
| Recurrence rate  (8 studies） | 5 | 0.19 | 0.09 | 0.42 | 3 | 1.50 | 0.63 | 3.60 | <0.01 |
| Clinical symptom improvement  (9studies） | 8 | — | — | — | 0 | — | — | — | NA |
| Satisfaction  (2studies） | 1 | — | — | — | 1 | — | — | — | NA |

**RR:** relative risk; **95%CI:** 95% confidence interval; **NA:** not applicable as no subgroup analysis was conducted for less than 2 studies in a given subgroup.

# eTable 6 Subgroup analysis of duration of surgery, hospital costs, volume of blood loss and hospital stay in patients treated with foam sclerotherapy vs. HL/S.

| Outcome | Non-English | | | | English | | | | **Test of interaction p-value** |
| --- | --- | --- | --- | --- | --- | --- | --- | --- | --- |
|  | No.  studies | WMD | 95%CI | | No.  studies | WMD | 95%CI | |  |
| Duration of surgery (min)  (15 studies） | 13 | -42.37 | -53.66 | -31.08 | 2 | 10.53 | -35.53 | 56.59 | 0.03 |
| Hospital costs (€)  (6 studies) | 4 | -463.27 | -487.06 | -429.48 | 2 | -849.99 | -1241.98 | -458.00 | 0.15 |
| Volume of blood loss (ml)  (9 studies) | 9 | — | — | — | 0 | — | — | — | NA |
| Hospital Stay (day)  (12 studies) | 12 | — | — | — | 0 | — | — | — | NA |

**WMD:** weighted mean difference; **CI:** confidence interval; **NA:** not applicable as no subgroup analysis was conducted for less than 2 studies in a given subgroup.

# eTable 7 Subgroup analysis of adverse events in GSV reflux patients treated with foam sclerotherapy vs.HL/S: Non-English or English

| Outcome | Non-English | | | | English | | | | **Test of interaction p-value** |
| --- | --- | --- | --- | --- | --- | --- | --- | --- | --- |
|  | No.  studies | RR | 95%CI | | No.  studies | RR | 95%CI | |  |
| AE-Pulmonary embolism  (5 studies) | 2 | 0.33 | 0.05 | 2.06 | 3 | 2.07 | 0.29 | 14.59 | 0.18 |
| AE-Infection  (8 studies) | 4 | 0.17 | 0.04 | 0.74 | 4 | 0.33 | 0.05 | 2.36 | 0.59 |
| AE-Phlebophlogosis  (9 studies) | 7 | 0.35 | 014 | 0.84 | 2 | 6.65 | 0.86 | 51.2 | 0.01 |
| AE-Hyperpigmentation  (11 studies) | 6 | 0.38 | 0.15 | 0.92 | 5 | 4.17 | 2.49 | 7.00 | <0.01 |
| AE-Lumpiness  (7 studies) | 5 | 0.33 | 0.15 | 0.75 | 2 | 2.43 | 1.13 | 5.21 | <0.01 |
| AE-Hematoma  (6 studies) | 4 | 0.31 | 0.09 | 1.00 | 2 | 0.33 | 0.20 | 0.53 | 0.80 |

**GSV:** great saphenous veins ; **RR:** relative risk ; **95%CI:** 95% confidence interval; **AE:** adverse events

# eTable 8 Subgroup analysis of adverse events in GSV reflux patients treated with foam sclerotherapy vs. HL/S with or without compression stockings

| Outcome | The treatment group wore socks | | | | Both groups wore socks | | | | **Test of interaction p-value** |
| --- | --- | --- | --- | --- | --- | --- | --- | --- | --- |
|  | No.  studies | RR | 95%CI | | No.  studies | RR | 95%CI | |  |
| AE- Phlebophlogosis  (4 studies) | 2 | 0.34 | 0.07 | 1.68 | 2 | 6.65 | 0.86 | 51.20 | 0.02 |
| AE-Hyperpigmentation  (8 studies) | 3 | 0.46 | 0.14 | 1.50 | 5 | 4.26 | 2.45 | 7.40 | <0.01 |

**GSV:** great saphenous veins; **AE:** adverse events; **RR:** relative risk; **95%CI:** 95% confidence interval

# eTable 9 Subgroup analysis of occlusion rate and recurrence rate among patients with GSV refux treated with foam sclerotherapy vs. HL/S stratified by follow-up duration.

| Outcome | Follow up ≤5 years | | | | Follow up ＞5 years | | | | **Test of interaction p-value** |
| --- | --- | --- | --- | --- | --- | --- | --- | --- | --- |
|  | No.  studies | RR | 95%CI | | No.  studies | RR | 95%CI | |  |
| Occlusion rate  (9 studies） | 6 | 0.78 | 0.65 | 0.94 | 3 | 0.50 | 0.31 | 0.81 | 0.09 |
| Recurrence rate  (6 studies） | 3 | 0.36 | 0.06 | 2.03 | 3 | 1.50 | 0.63 | 3.60 | 0.15 |

# eTable 10 ICEMAN criteria or assessing the credibility of subgroup effects

| Criteria | Subgroup effects | | | | |
| --- | --- | --- | --- | --- | --- |
|  | Duration of surgery and English vs.Non-English publication | AE-Phlebophlogosis  and English vs. Non-English publication | AE-Hyperpigmentation and English vs.Non-English publication | AE-Phlebophlogosis  and the treatment group wore socks vs.both groups wore socks | AE-Hyperpigmentation  and the treatment group wore socks vs.both groups wore socks |
| 1: Is the analysis of effect modification based on comparison within rather than between trials? | Completely Between-study | Completely Between-study | Completely Between-study | Completely Between-study | Completely Between-study |
| 2: For within-trial comparisons, is the effect modification similar from trial to trial? | Not applicable | Not applicable | Not applicable | Not applicable | Not applicable |
| 3: For between-trial comparisons, is the number of trials large? | Very small  (2 studies with English ) | Rather small  (3 studies with English) | Rather small  (5 studies with English) | Very small  (2 studies with the treatment group wore socks) | Rather small  (3 studies with the treatment group wore socks) |
| 4: Was the direction of effect modification correctly hypothesized a priori? | Probably no | Probably yes | Probably yes | Probably yes | Probably yes |
| 5: Does a test for interaction suggest that chance is an unlikely explanation of the apparent effect modification? | Chance a likely explanation or unclear (p≤0.05 and＞0.01 ) | Chance may not explain (p=0.01) | Chance may not explain (p=≤0.01 and >0.005) | Chance a likely explanation or unclear (p≤0.05 and＞0.01 ) | Chance may not explain (p≤0.01 and >0.005) |
| 6: Did the authors test only a small number of effect modifiers or consider the number in their statistical analysis? | Probably no (2 factors) | Probably no (2 factors) | Probably no (2 factors) | Probably no (2 factors) | Probably no (2 factors) |
| 7: Did the authors use a random effects model? | Definitely yes | Definitely yes | Definitely yes | Definitely yes | Definitely yes |
| 8: If the effect modifier is a continuous variable, were arbitrary cut points avoided? | NA | NA | NA | NA | NA |
| 9: Optional: Are there any additional considerations that may increase or decrease credibility? | No | No | No | No | No |
| The effect modification persisted after adjustment for other potential effect modifiers | NA | NA | NA | NA | NA |
| The effect modification is consistent across related outcomes: | No | No | No | No | No |
| A sensitivity analysis suggested robustness to relevant assumptions | NA | NA | NA | NA | NA |
| Effect modification supported by external evidence | NA | NA | NA | NA | NA |
| “Dose-response effect” across levels of the effect modifier | No | NA | NA | NA | NA |
| Risk of bias of the main effects of the individual RCTs or the meta-analysis | No | No | No | No | No |
| The meta-analysis had had exceptionally high power to detect the effect modification | NA | NA | NA | NA | NA |
| Overall credibility | Very low | Low | Low | Low | Low |

NA: not applicable

# eTable 11 Sensitivity analysis for binary outcomes

| **Outcomes** | **Sensitivity analysis** | **No. of**  **studies** | **Total**  **events (n)** | **Total sample size**  **(N)** | **RR** | **95%CI** | |
| --- | --- | --- | --- | --- | --- | --- | --- |
| **Occlusion rate** | Primary Analysis (D-L method) | 6 | 768 | 1164 | 0.65 | 0.49 | 0.86 |
|  | HKSJ method | 6 | 768 | 1164 | 0.64 | 0.44 | 0.93 |
|  | Single treatment | 4 | 413 | 651 | 0.58 | 0.37 | 0.92 |
| **Recurrence rate** | Primary Analysis (D-L method) | 8 | 207 | 966 | 0.56 | 0.25 | 1.23 |
|  | HKSJ method | 8 | 207 | 966 | 0.50 | 0.16 | 1.54 |
|  | Single treatment | 7 | 123 | 739 | 0.41 | 0.15 | 1.10 |
| **Symptom improvement** | Primary Analysis (D-L method) | 9 | 672 | 736 | 1.09 | 1.03 | 1.16 |
|  | HKSJ method | 9 | 672 | 736 | 1.11 | 1.02 | 1.20 |
| AE-pulmonary embolism | Primary Analysis (D-L method) | 5 | 7 | 980 | 0.78 | 0.21 | 2.95 |
|  | HKSJ method | 5 | 7 | 980 | 0.80 | 0.19 | 3.37 |
|  | Single treatment | 3 | 6 | 405 | 0.57 | 0.12 | 2.76 |
| AE-Paraesthesia | Primary Analysis (D-L method) | 4 | 19 | 955 | 0.44 | 0.15 | 1.27 |
|  | HKSJ method | 4 | 19 | 955 | 0.42 | 0.11 | 1.55 |
|  | Single treatment | 2 | 11 | 380 | 0.55 | 0.16 | 1.89 |
| AE-Infection | Primary Analysis (D-L method) | 8 | 25 | 1283 | 0.28 | 0.10 | 0.76 |
|  | HKSJ method | 8 | 25 | 1283 | 0.26 | 0.08 | 0.81 |
|  | Single treatment | 6 | 16 | 610 | 0.15 | 0.05 | 0.51 |
| AE-phlebothrombosis | Primary Analysis (D-L method) | 4 | 11 | 898 | 0.76 | 0.25 | 2.34 |
|  | HKSJ method | 4 | 11 | 898 | 0.78 | 0.23 | 2.59 |
|  | Single treatment | 2 | 3 | 323 | 0.61 | 0.08 | 4.88 |
| AE-Thrombophlebitis | Primary Analysis (D-L method) | 10 | 66 | 1267 | 0.71 | 0.26 | 1.92 |
|  | HKSJ method | 10 | 66 | 1267 | 0.70 | 0.25 | 1.98 |
|  | Single treatment | 9 | 49 | 837 | 0.56 | 0.23 | 1.39 |
| AE-Hyperpigmentation | Primary Analysis (D-L method) | 11 | 222 | 1924 | 1.51 | 0.70 | 3.28 |
|  | HKSJ method | 11 | 222 | 1924 | 1.39 | 0.57 | 3.36 |
|  | Single treatment | 9 | 207 | 1389 | 1.20 | 0.49 | 2.94 |
| AE-Haematoma | Primary Analysis (D-L method) | 6 | 67 | 893 | 0.27 | 0.16 | 0.44 |
|  | HKSJ method | 6 | 67 | 893 | 0.27 | 0.16 | 0.45 |
|  | Single treatment | 5 | 64 | 463 | 0.27 | 0.17 | 0.45 |
| AE-Skin ulceration | Primary Analysis (D-L method) | 2 | 4 | 567 | 0.97 | 0.19 | 4.84 |
|  | HKSJ method | 2 | 4 | 567 | 0.96 | 0.04 | 20.88 |
| AE-Numbness | Primary Analysis (D-L method) | 7 | 76 | 945 | 0.25 | 0.14 | 0.42 |
|  | HKSJ method | 7 | 76 | 945 | 0.24 | 0.13 | 0.45 |
| AE-Lumpiness | Primary Analysis (D-L method) | 7 | 201 | 1010 | 0.93 | 0.42 | 2.02 |
|  | HKSJ method | 7 | 201 | 1010 | 0.81 | 0.32 | 2.05 |
| AE-Subcutaneous stasis | Primary Analysis (D-L method) | 7 | 102 | 969 | 0.61 | 0.33 | 1.13 |
|  | HKSJ method | 7 | 102 | 969 | 0.49 | 0.21 | 1.14 |

**AE:**adverse events; **RR:** relative risk；**CI:** 95% confidence interval

# eTable 12 Sensitivity analysis for continuous outcomes

| **Outcomes** | **Sensitivity analysis** | **No. of**  **studies** | **Total sample size**  **(N)** | **WMD** | **95%CI** | |
| --- | --- | --- | --- | --- | --- | --- |
| Hospital costs（€） | Primary Analysis (D-L method) | 6 | 1263 | -4622.34 | -6340.55 | -2904.14 |
|  | HKSJ method | 6 | 1263 | -4683.37 | -6166.62 | -3200.11 |
|  | Only one treatment | 5 | 833 | -3969.94 | -4702.70 | -3237.17 |
| Duration of surgery (min) | Primary Analysis (D-L method) | 15 | 1843 | -35.28 | -45.80 | -24.75 |
|  | HKSJ method | 15 | 1843 | -35.54 | -56.07 | -15.02 |
|  | Only one treatment | 14 | 1595 | -36.88 | -47.88 | -25.88 |
| VCSS (score ) | Primary Analysis (D-L method) | 3 | 1105 | -0.20 | -0.45 | 0.05 |
|  | HKSJ method | 3 | 1105 | -0.20 | -0.46 | 0.06 |

**W**

**WMD:** Weighted mean difference; **CI:** 95% confidence inter

# eFigure . 1 Risk of bias assessment of 23 eligible randomized controlled trials

# eFigure . 2 Symptom improvement of GSV reflux treated with foam sclerotherapy vs. HL/S.

# eFigure . 3 Duration of surgery after foam sclerotherapy vs. HL/S treatment of GSV reflux.

**GSV:** great saphenous veins ; **CI:** 95% confidence interval

eFigure . 4 Hospital stay after foam sclerotherapy vs. HL/S treatment of GSV reflux.

**GSV:** great saphenous veins ; **CI:** 95% confidence interval

eFigure . 5 Hospital costs after foam sclerotherapy vs. HL/S treatment of GSV reflux.

**GSV:** great saphenous veins ; **CI:** 95% confidence interval

eFigure . 6 Volume of blood loss after foam sclerotherapy vs. HL/S treatment of GSV reflux.

**GSV:** great saphenous veins ; **CI:** 95% confidence interval

eFigure . 7 VCSS score after foam sclerotherapy vs. HL/S treatment of GSV reflux.

**VCSS：**Venous Clinical Severity Score; **GSV:** great saphenous veins ; **95%CI:** 95% confidence interval

eFigure . 8 Quality of life after foam sclerotherapy vs. HL/S treatment of GSV reflux.

**GSV:** great saphenous veins ; **CI:** 95% confidence interval

eFigure . 9 Patient satisfaction after foam sclerotherapy vs. HL/S treatment of GSV reflux.

**GSV:** great saphenous veins ; **95% CI:** 95% confidence interval

# eFigure . 10 Incidence of infection after foam sclerotherapy vs. **HL/S** treatment of GSV reflux.

**AE:** adverse events; **GSV:** great saphenous veins ; **95% CI:** 95% confidence interval

# eFigure . 11 Incidence of numbness after foam sclerotherapy vs. **HL/S** treatment of GSV reflux.

**AE:** adverse events; **GSV:** great saphenous veins ; **95% CI:** 95% confidence interval

# eFigure . 12 Incidence of haematoma after foam sclerotherapy vs. **HL/S** treatment of GSV reflux.

**AE:** adverse events; **GSV:** great saphenous veins ; **95% CI:** 95% confidence interval

eFigure . 13 Incidence of pulmonary embolism after foam sclerotherapy vs. HL/S treatment of GSV reflux.**AE:** adverse events; **GSV:** great saphenous veins ; **95% CI:** 95% confidence interval

# eFigure . 14 Incidence of paraesthesia after foam sclerotherapy vs. HL/S treatment of GSV reflux.

**AE:** adverse events;**GSV:** great saphenous veins ; **95% CI:** 95% confidence interval

# eFigure . 15 Incidence of phlebothrombosis after foam sclerotherapy vs.**HL/S** treatment of GSV reflux.

**AE:** adverse events; **GSV:** great saphenous veins ; **95% CI:** 95% confidence interval

# eFigure . 16 Incidence of thrombophlebitis after foam sclerotherapy vs. **HL/S** treatment of GSV reflux.

**AE:** adverse events; **GSV:** great saphenous veins ; **95% CI:** 95% confidence interval

# eFigure . 17 Incidence of skin ulceration after foam sclerotherapy vs. **HL/S** treatment of GSV reflux.

**AE:** adverse events; **GSV:** great saphenous veins; **95% CI:** 95% confidence interval

# eFigure . 18 Incidence of lumpiness after foam sclerotherapy vs. **HL/S** treatment of GSV reflux.

**AE:** adverse events; **GSV:** great saphenous veins; **95% CI:** 95% confidence interval

# eFigure . 19 Incidence of subcutaneous stasis after foam sclerotherapy vs. **HL/S** treatment of GSV reflux.

**AE:** adverse events; **GSV:** great saphenous veins ; **95% CI:** 95% confidence interval

# eFigure . 20 Incidence of hyperpigmentation after foam sclerotherapy vs. **HL/S** treatment of GSV reflux.

**AE:** adverse events; **GSV:** great saphenous veins ; **95% CI:** 95% confidence interval

# eFigure . 21 Incidence of nerve injury after foam sclerotherapy vs.**HL/S** treatment of GSV reflux.

**AE:** adverse events; **GSV:** great saphenous veins ; **95% CI:** 95% confidence interval

# eFigure . 22 Subgroup Analysis: Comparison of Recurrence Rates Between foam sclerotherapy and HL/S for GSV reflux in Non-English versus English Publications

**GSV:** great saphenous veins ; **95% CI:** 95% confidence interval

Alt text: This subgroup analysis compared recurrence rates between foam sclerotherapy and HL/S for GSV reflux, stratified by Non-English vs. English language publications.

# eFigure . 23 Subgroup Analysis: Comparison of Duration of surgery Between foam sclerotherapy and HL/S for GSV reflux in Non-English versus English Publications

**GSV:** great saphenous veins ; **95% CI:** 95% confidence interval

Alt text: This subgroup analysis compared surgery duration between foam sclerotherapy and HL/S for GSV reflux, stratified by Non-English vs. English language publications.

# eFigure . 24 Subgroup Analysis: Comparison of Hospital Costs Between foam sclerotherapy and HL/S for GSV reflux in Non-English versus English Publications

**GSV:** great saphenous veins ; **95% CI:** 95% confidence interval

Alt text: This subgroup analysis compared hospitalization costs between foam sclerotherapy (treatment) and HL/S (control) for GSV reflux, stratified by Non-English vs. English language publications.

# eFigure . 25 Subgroup Analysis: Comparison of Adverse Event Pulmonary Embolism Between foam sclerotherapy and HL/S for GSV reflux in Non-English versus English Publications

**AE:** adverse events; **GSV:** great saphenous veins ; **95% CI:** 95% confidence interval

Alt text: This subgroup analysis compared pulmonary embolism (PE) adverse events between foam sclerotherapy and HL/S for GSV reflux, stratified by Non-English vs. English language publications.

# eFigure . 26 Subgroup Analysis: Comparison of Adverse Event Infection Between foam sclerotherapy and HL/S for GSV reflux in Non-English versus English Publications

**AE:** adverse events; **GSV:** great saphenous veins; **95% CI:** 95% confidence interval

Alt text: This subgroup analysis compared infection adverse events between foam sclerotherapy and HL/S for GSV reflux, stratified by Non-English vs. English language publications.

# eFigure . 27 Subgroup Analysis: Comparison of Adverse Event Phlebophlogosis Between foam sclerotherapy and HL/S for GSV reflux in Non-English versus English Publications

**GSV:** great saphenous veins; **95% CI:** 95% confidence interval

Alt text: This subgroup analysis compared phlebophlogosis adverse events between foam sclerotherapy and HL/S for GSV reflux, stratified by Non-English vs. English language publications.

# eFigure . 28 Subgroup Analysis: Comparison of Adverse Event Lumpiness Between foam sclerotherapy and HL/S for GSV reflux in Non-English versus English Publications

**AE:** adverse events; **GSV:** great saphenous veins; **95% CI:** 95% confidence interval

Alt text: This subgroup analysis compared lumpiness adverse events between foam sclerotherapy and HL/S for GSV reflux, stratified by Non-English vs. English language publications.

# eFigure . 29 Subgroup Analysis: Comparison of Adverse Event Hyperpigmentation Between foam sclerotherapy and HL/S for GSV reflux in Non-English versus English Publications

**AE:** adverse events; **GSV:** great saphenous veins; **95% CI:** 95% confidence interval

Alt text: This subgroup analysis compared hyperpigmentation adverse events between foam sclerotherapy and HL/S for GSV reflux, stratified by Non-English vs. English language publications.

# eFigure . 30 Subgroup Analysis: Comparison of Adverse Event Hematoma Between foam sclerotherapy and HL/S for GSV reflux in Non-English versus English Publications

**AE:** adverse events;**GSV:** great saphenous veins; **95% CI:** 95% confidence interval

Alt text: This subgroup analysis compared hematoma adverse events between foam sclerotherapy and HL/S for GSV reflux, stratified by Non-English vs. English language publications.

# eFigure . 31 Subgroup Analysis: Comparison of Adverse Event Thrombophlebitis Between foam sclerotherapy and HL/S for GSV reflux in Treatment Group Wore Socks versus Both Groups Wore Socks.

**GSV:** great saphenous veins; **95% CI:** 95% confidence interval

Alt text: This subgroup analysis compared thrombophlebitis adverse events between foam sclerotherapy and HL/S for GSV reflux, stratified by sock-wearing protocols.

# eFigure . 32 Subgroup Analysis: Comparison of Adverse Event Hyperpigmentation Between foam sclerotherapy and HL/S for GSV reflux in Treatment Group Wore Socks versus Both Groups Wore Socks.

**GSV:** great saphenous veins; **95% CI::** 95% confidence interval

Alt text: This subgroup analysis compared hyperpigmentation adverse events between foam sclerotherapy and HL/S for GSV reflux, stratified by whether only the treatment group wore socks or both groups wore socks.

# eFigure . 33 Subgroup Analysis: Comparison of Occlusion Rates Between foam sclerotherapy and HL/S for GSV reflux in Follow up ≤ 5 years versus Follow up ＞ 5 years

\

**GSV:** great saphenous veins; **95% CI::** 95% confidence interval

# eFigure . 34 Subgroup Analysis: Comparison of Recurrence Rates Between foam sclerotherapy and HL/S for GSV reflux in Follow up ≤ 5 years versus Follow up ＞ 5 years

**GSV:** great saphenous veins; **95% CI::** 95% confidence interval

# Search strategies

| Database | Search strategies |
| --- | --- |
| **Cochrane** | #1 (((varicose* OR saphenous) AND vein*) OR "venous insufficie  ncy*" OR varicos*):ti,ab,kw  #2 MeSH descriptor: [Varicose Veins] explode all trees  #3 #1 or #2  #4 ("Sclerotherapy" OR sclero* OR (tetradecyl AND (sulfate OR sulphate)) OR "Saline Solution" OR Ethanolamines OR (polydocanol or polidocanol) OR "ethanolamine oleate" OR "sodium morrhuate" OR sotradecol OR (aetoxisclerol or aethoxysclerol) OR (aetoxiskerol or aethoxyskerol)):ti,ab,kw  #5 MeSH descriptor: [Sclerotherapy] explod trees  #6 #4 or #5  #7 #3 and #6  #8 randomized controlled trial:pt OR controlled clinical trial:pt OR randomized: ti,ab OR placebo:ti,ab OR randomly:ti,ab OR trial:ti,ab  #9 MeSH descriptor: [animals] explode all trees  #10 MeSH descriptor: [humans] explode all trees  #11 #9 not #10  #12 #8 not #11  #13 #7 and #12 |
| **EMBASE** | #1 'Sclerotherapy'/exp or 'Sclerosing Solutions'/exp or 'Sodium Tetradecyl Sulfate'/exp or 'Saline Solution, Hypertonic'/exp  #2 sclerotherapy* :ti,ab,kw OR ligation*:ti,ab,kw OR sclerotherap* :ti,ab,kw OR strip* :ti,ab,kw OR avulsion*:ti,ab,kw  #3 #1 or #2  #4 'Varicose Veins'/exp or 'Venous Insufficiency'/exp or 'Saphenous Vein'/exp  #5 ("Sclerotherapy" OR sclero* OR (tetradecyl AND (sulfate OR sulphate)) OR "Saline Solution" OR Ethanolamines OR (polydocanol or polidocanol) OR "ethanolamine oleate" OR "sodium morrhuate" OR sotradecol OR (aetoxisclerol or aethoxysclerol) OR (aetoxiskerol or aethoxyskerol)):ti,ab,kw  #6 #4 or #5  #7 #3 and #6  #8 search filter for rct  #9 #7 and  #10 letter:it or comment:it or editorial:it or review:it  #11 #9 not #10  RCT filter:  1 ‘randomized controlled trial ’/exp  2 ‘controlled clinical trial ’/exp  3 random*:ti,ab,tt  4 ‘randomization ’/exp  5 ‘intermethod comparison ’/exp  6 placebo:ti,ab,t  7 (compare:ti,tt OR compared:ti,tt OR comparison:ti,tt)  8 ((evaluated:ab OR evaluate:ab OR evaluating:ab OR assessed:ab OR assess:ab) AND (compare:ab OR compared:ab OR comparing:ab OR comparison:ab))  9 (open NEXT/1 label):ti,ab,tt  10 ((double OR single OR doubly OR singly) NEXT/1 (blind OR blinded OR blindly)):ti,ab,tt  11 ‘double blind procedure ’/exp  12 (parallel NEXT/1 group*):ti,ab,tt  13 (crossover:ti,ab,tt OR ‘cross over ’:ti,ab,tt)  14 ((assign* OR match OR matched OR allocation) NEAR/6 (alternate OR group OR groups OR intervention OR interventions OR patient OR patients OR subject OR subjects OR participant OR participants)):ti,ab,tt  15 (assigned:ti,ab,tt OR allocated:ti,ab,tt)  16 (controlled NEAR/8 (study OR design OR trial)):ti,ab,tt  17 (volunteer:ti,ab,tt OR volunteers:ti,ab,tt)  18 ‘human experiment ’/exp  19 trial:ti,tt  20 #1 OR #2 OR #3 OR #4 OR #5 OR #6 OR #7 OR #8 OR #9 OR #10 OR #11 OR #12 OR #13 OR #14 OR #15 OR #16 OR #17 OR #18 OR #19  21 (((random* NEXT/1 sampl* NEAR/8 ( ‘ cross section* ’ OR questionnaire* OR survey OR surveys OR database or databases)):ti,ab,tt) NOT (‘comparative study ’/exp OR‘controlled study ’ /exp OR ‘randomised controlled ’:ti,ab,tt OR ‘randomized controlled ’:ti,ab,tt OR ‘randomly assigned ’:ti,ab,tt))  22 (‘cross ‐sectional study ’/exp NOT (‘randomized controlled trial ’/exp OR ‘controlled clinical study ’/exp OR ‘controlled study ’/exp OR ‘randomised controlled ’:ti,ab,tt OR ‘randomized controlled ’:ti,ab,tt OR ‘control group ’:ti,ab,tt OR ‘control groups ’:ti,ab,tt))  23 (‘case control* ’:ti,ab,tt AND random*:ti,ab,tt NOT (‘ randomised controlled ’:ti,ab,tt OR ‘randomized controlled ’:ti,ab,tt))  24 (‘systematic review ’:ti,tt NOT (trial:ti,tt OR study:ti,tt))  25 (nonrandom*:ti,ab,tt NOT random*:ti,ab,tt)  26 ‘random field* ’:ti,ab,tt  27 (‘random cluster ’ NEAR/4 sampl*):ti,ab,tt  28 (review:ab AND review:it) NOT trial:ti,tt  29 (‘we searched ’:ab AND (review:ti,tt OR review:it))  30 ‘update review ’:ab  31 (databases NEAR/5 searched):ab  32 ((rat:ti,tt OR rats:ti,tt OR mouse:ti,tt OR mice:ti,tt OR swine:ti,tt OR porcine:ti,tt OR murine:ti,tt OR sheep:ti,tt OR lambs:ti,tt OR pigs:ti,tt OR piglets:ti,tt OR rabbit:ti,tt OR rabbits:ti,tt OR cat:ti,tt OR cats:ti,tt OR dog:ti,tt OR dogs:ti,tt OR cattle:ti,tt OR bovine:ti,tt OR monkey:ti,tt OR monkeys:ti,tt OR trout:ti,tt OR marmoset*:ti,tt) AND ‘animal experiment ’/exp)  33 (‘animal experiment ’/exp NOT (‘human experiment ’/exp OR ‘human ’/exp))  34 #21 OR #22 OR #23 OR #24 OR #25 OR #26 OR #27 OR #28 OR #29 OR #30 OR #31 OR #32 OR #33  35 #20 NOT #34  #36 'Sclerotherapy'/exp or 'Sclerosing Solutions'/exp or 'Sodium Tetradecyl Sulfate'/exp or 'Saline Solution, Hypertonic'/exp  #37 ("Sclerotherapy" OR sclero* OR (tetradecyl AND (sulfate OR sulphate)) OR "Saline Solution" OR Ethanolamines OR (polydocanol or polidocanol) OR "ethanolamine oleate" OR "sodium morrhuate" OR sotradecol OR (aetoxisclerol or aethoxysclerol) OR (aetoxiskerol or aethoxyskerol)):ti,ab,kw  #38 #36 or #37  #39 'Varicose Veins'/exp or 'Venous Insufficiency'/exp or 'Saphenous Vein'/exp  #40 (((varicose* OR saphenous vein*) AND vein*) OR "venous insufficiency*" OR varicos* ):ti,ab,kw  #41 #39 or #40  #42 #35 and #38 and #41  #43 letter:it or comment:it or editorial:it or review:it  #44 #42 not #43 |
| **Pubmed** | #1 "Varicose Veins" [Mesh] OR ((varicose*[Title/Abstract] OR saphenous[Title/Abstract]) AND vein*[Title/Abstract]) OR "venous insufficiency*"[Title/Abstract] OR varicos*[Title/Abstract]  #2 "Sclerotherapy" [Mesh] OR sclero*[Title/Abstract] OR (tetradecyl[Title/Abstract] AND (sulfate[Title/Abstract] OR sulphate[Title/Abstract])) OR "Saline Solution" [Title/Abstract] OR Ethanolamines[Title/Abstract] OR (polydocanol or polidocanol[Title/Abstract]) OR "ethanolamine oleate" [Title/Abstract] OR "sodium morrhuate" [Title/Abstract] OR sotradecol[Title/Abstract] OR (aetoxisclerol or aethoxysclerol[Title/Abstract]) OR (aethoxyskerol[Title/Abstract])  #3 randomized controlled trial [pt] OR controlled clinical trial [pt] OR randomized [tiab] OR placebo [tiab] OR clinical trials as topic [mesh:noexp] OR randomly [tiab] OR trial [ti]  #4 animals [mh] NOT humans [mh]  #5 #3 NOT #4  #6 #1 AND #2 AND #5  #7 “ letter ”[publication type] OR“ comment ”[publication type] OR “ editorial ”  [publication type] OR review [publication type]  #8 #6 NOT #7 |
| **Web of science** | #1 TS=("Varicose Veins" OR ((varicose* OR saphenous) AND vein*) OR "venous insufficiency*" OR varicos*)  #2 TS=("Sclerotherapy" OR sclero* OR (tetradecyl AND (sulfate OR sulphate)) OR "Saline Solution" OR Ethanolamines OR (polydocanol or polidocanol) OR "ethanolamine oleate" OR "sodium morrhuate" OR sotradecol OR (aetoxisclerol or aethoxysclerol) OR (aetoxiskerol or aethoxyskerol))  #3 TS= clinical trial* OR (TS=comparative stud* OR TS=controlled trial* OR TS=follow-up stud* OR TS=prospective stud* OR TS=random* OR TS=placebo* OR TS=single blind* OR TS=double blind*)  #4 #1 AND #2 AND #3  #5 (#1 AND #2 AND #3) and Review or Meeting or Case Report or Editorial or Book or Letter |
| **CNKI** | (TKA= '硬化溶液' OR TKA= '硬化疗法' OR TKA= '硬化剂' OR TKA= '泡沫硬化剂' OR TKA= '聚多卡 醇' OR TKA= '鱼肝油酸钠' OR TKA= '乙醇胺油酸酯' OR TKA= '十四烷基硫酸钠' OR TKA= '泡沫 硬化术') AND (TKA= '静脉曲张' OR TKA= '下肢静脉曲张' OR TKA= '大隐静脉' OR TKA= '曲张') AND (SU %= '随机' OR SU %= '对照') |
| **Wanfang** | 主题: ("硬化溶液" OR "硬化疗法" OR "硬化剂" OR "泡沫硬化剂" OR "'聚多卡醇" OR "鱼肝油 酸钠" OR "乙醇胺油酸酯" OR "十四烷基硫酸钠" OR "泡沫硬化术") AND 主题: ("静脉曲张" OR "下肢静脉曲张" OR "大隐静脉" OR "曲张") AND 主题: ("随机" OR "对照") |
| **CQVIP** | M=(硬化溶液 OR 硬化疗法OR 硬化剂OR 泡沫硬化剂 OR 聚多卡醇 OR 鱼肝油酸钠 OR 乙醇胺油酸酯OR 十四烷基硫酸钠 OR 泡沫硬化术) AND M=(静脉曲张 OR 下肢静脉曲张 OR 大隐静脉OR 曲张) AND U=(随机 OR 对照) |
| **CBM** | ("静脉曲张"[不加权:扩展] OR "脉管曲张"[常用字段:智能] OR "静脉曲张"[常用字段:智能] OR "下肢静脉曲张"[常用字段:智能] OR "大隐静脉"[常用字段:智能] OR "曲张"[常用字段:智能]) AND ("硬化疗法"[不加权:扩展] OR "硬化溶液"[不加权:扩展] OR "硬化溶液"[不加权:扩展] OR "硬化疗法"[常用字段:智能]) AND ("随机对照试验(主题)" [不加权:扩展] OR "随机"[常用字段: 智能] OR "对照"[常用字段:智能]) |
